# Altered Mitochondrial Metabolism and Mechanosensation in the Failing Heart: Focus on Intracellular Calcium Signaling

**DOI:** 10.3390/ijms18071487

**Published:** 2017-07-10

**Authors:** Aderville Cabassi, Michele Miragoli

**Affiliations:** 1 Department of Medicine and Surgery, University of Parma, 43126 Parma, Italy; aderville.cabassi@unipr.it; 2 Institute of Genetic and Biomedical Research, National Research Council, 20138 Milan, Italy

**Keywords:** mechanoelectric transduction, mitochondria metabolism, calcium transient, microdomains, fatty acids

## Abstract

The heart consists of millions of cells, namely cardiomyocytes, which are highly organized in terms of structure and function, at both macroscale and microscale levels. Such meticulous organization is imperative for assuring the physiological pump-function of the heart. One of the key players for the electrical and mechanical synchronization and contraction is the calcium ion via the well-known calcium-induced calcium release process. In cardiovascular diseases, the structural organization is lost, resulting in morphological, electrical, and metabolic remodeling owing the imbalance of the calcium handling and promoting heart failure and arrhythmias. Recently, attention has been focused on the role of mitochondria, which seem to jeopardize these events by misbalancing the calcium processes. In this review, we highlight our recent findings, especially the role of mitochondria (dys)function in failing cardiomyocytes with respect to the calcium machinery.

## 1. Introduction

The heart pumps more than 60 times per minute, it is an exquisite electromechanical organ, where every contraction is activated and triggered with the preceding bioelectrical stimulus. 

The electro-mechanical coupling can also be reversible whereby a mechanical insult or stretch can generate bioelectricity via the well-known phenomena of mechanoelectric feedback [1,2].

Mechanoelectric feedback fine-tunes heart contractions so that every single heartbeat can be adjusted in response to the previous contraction. When this mechanosensation-based tuning is lost, arrhythmias and electrical instability occur [3].

Several colleagues have investigated mechanoelectric feedback in large animals and at the cellular level; however, due to the lack of adequate technologies, subcellular study is a very recent matter of investigation [4,5,6]. They found mechanosensation related to sarcomeric components [7] but mainly proteins deputed to regulate signal functions [8,9]. They also found that mutations in complex proteins (mainly in Z-grooves) could cause abnormal intracellular Ca^2+^ signaling [9]. The ‘cardiac remodeling’ involves cell structures (cardiomyocytes, fibroblasts, cell membrane, organelles, nuclei, cytoskeleton, etc.) [10,11,12,13,14] and bioelectric counterparts such as ion channels and pump [15,16], receptors [17], impulse initiation [18], and signalling pathways [19].

Cardiac remodeling can be also metabolic during heart failure progression and aging [20,21]. Recently, a pivotal role has emerged from an organelle deputed to controlling cell metabolism and respiration: the mitochondria.

### Mitochondria in Cardiac Disease: Do We Need to Study Them?

Mitochondria, subsarcolemmal or perinuclear [22], are organelles involved in controlling cardiac metabolism. Cardiac pathologies (arrhythmia or ischemia) are frequently associated with energy decreases [22,23]. Mitochondrial biogenesis is enhanced during the cardiomyocyte compensated hypertrophy phase in an effort to match the increased energy demand, but is subsequently decreased when the decompensation phase of heart failure emerges [24]. There are mitochondrial subpopulations that are impacted differentially in heart failure; in fact, those located within the contractile apparatus (interfibrillar mitochondria) are less affected in volume overload heart failure models whereas those located under the sarcolemma seem to be primarily affected [25]. The location of mitochondria is also important. For example, during myocardial infarction [26] the regular alignment is lost and we know that this is correlated with maintaining the homeostasis of excitation–contraction coupling [5,27,28] and, of course, intracellular calcium activation [29]. Intracellular calcium levels represent a key signaling messenger to mitochondria with changes leading to the maintenance of life signals or activation of programmed death pathways [30].

These metabolic changes occurring in ischemic and failing heart are also directly or indirectly participating in the arrhythmogenic processes [22,23]. We have minimal information regarding mitochondrial function in the human heart as most studies are focused on animal models or single cell investigations which reflect only in part what happens in the 3D human cardiac environment. However, cardiac surgeries, such as atrium cannulation and ventricular biopsies, provide a source of fresh tissue, which is underused for research on mitochondrial pathophysiology. One has to keep in mind that the heart is the organ, which houses the highest mitochondrial density (35% vs. 3–8% of mitochondria in smooth muscle cells) of any organ as the main energy production for cardiac contraction comes from oxidative phosphorylation. Based on these findings, we have to consider mitochondrial dysfunction as a direct process in cardiac failure knowing that such organelles supply the energy demanded by the cardiac tissue [24].

Cardiac pathophysiological remodeling includes mitochondrial function [31,32] and metabolism as observed in aging and failing hearts, and in particular during the development of various cardiovascular diseases when compared to cardiac energetics of a healthy person. In fact, cardiac damages, caused by ischemia, infarction, hypertrophy/dilation due to pressure, and volume overloads are characterized by cardiac structural and metabolic remodeling, all progressing to heart failure [33,34,35].

Hence, the following questions emerged: does the alteration of microdomain-related mitochondrial alignment play a role in the genesis of arrhythmia? Is mitochondrial metabolism involved? The answer to these questions are thought-provoking as (i) the role of mitochondria in heart failure is probably underestimated and (ii) many conventional technologies cannot selectively activate or interrogate mitochondrial mechanosensitivity at the sarcolemmal microdomain level.

## 2. Altered Mitochondria Metabolism in Heart Failure and Consequences on Calcium Handling

Whatever the underlying cause of heart failure—ischemic, hypertensive, or idiopathic dilated cardiomyopathy—this highly prevalent disease, once present, progresses slowly, even if current medical therapy appears to be able to slow down its exacerbations and above all to improve its mortality [36]. Changes in patients or in experimental models of heart failure (HF) are observable in the course of progression from the normal heart to the failing heart including cardiac remodeling [37]. The progression of HF as well as the complication of this clinical condition clearly include mitochondrial remodeling in terms of energy production, calcium handling, and reactive oxygen or nitrogen species generation [31,32,38]. Fatty acid (FA) is the main substrate for cardiac energetics in normal heart; they are responsible for four-fifths of myocardial generated ATP, whilst the remaining part is related to glucose oxidation.

**Figure 1:**
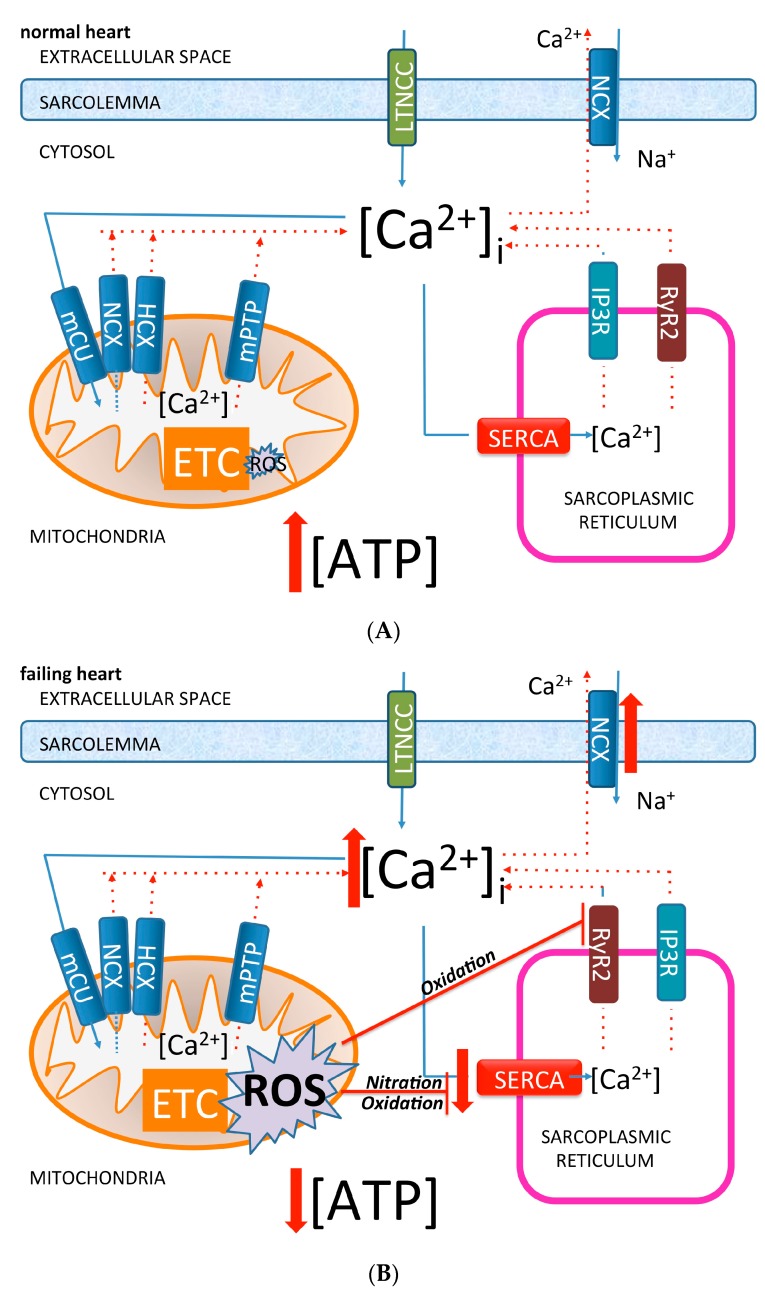
Diagram of subcellular interaction between subcellular calcium compartmentation and reactive oxygen species (ROS) in normal (**A**) and failing (**B**) cardiomyocytes. LTCC: L-Type Calcium Channel; NCX: Sodium-Calcium Exchanger; HCX: Hydrogen-Calcium Exchanger; mCU: Mitochondria Calcium Uniporter; mPTP: Mitochondrial Permeability Transition Pore; SERCA: Sarco/Endoplasmic Reticulum Ca^2+^-ATPase; IP3R: Inositol Trisphosphate Receptors; RyR: Ryanodine Receptors; ROS: Reactive Oxygen Species; ETC: Electron Transport Chain. Dotted lines indicate Ca^2+^ efflux; full line indicate Ca^2+^ influx.

Even if fatty acids generate more ATP than glucose, this process needs more oxygen and therefore ATP production to oxygen consumption ratio is higher than that for glucose utilization. Elevated FA oxidation reduces cardiac efficiency. Fatty acid beta-oxidation rise was seen as an attempt to compensate for increased energy demand in cardiomyocytes in the early phases of heart failure development [39]. In advanced heart failure settings, FA beta-oxidation and mitochondrial respiratory activity decrease leading to decrease cardiac ATP generation (Figure 1A,B). Glucose uptake and glycolysis are instead upregulated but are not able to compensate for the loss in ATP generation due to the marked reduction of FA oxidation [39].

The integrity of mitochondrial energetics allows fine calcium regulation and tuning by cardiomyocytes at the subcellular level. Appropriate calcium handling is necessary not just for efficient mitochondrial phosphorylation but also for keeping the reactive oxygen and nitrogen species (ROS and RNS) generation under close control (c.f. paragraphs below) [33,40]. In a physiological condition, oxidative phosphorylation pathways generate a small amount (1–2%) of electrons leaking from electron transfer chain to form ROS. Such a quantity is markedly amplified in HF [41,42]. Almost 70 percent of mitochondria-generated ATP is consumed for cardiac contractions and the rest for ion pump functioning. A major portion of ATP consumed is related to sarcoplasmic reticulum Ca^2+^-ATPase (SERCA) [43] (Figure 1A,B).

Mitochondrial calcium handling is fundamental in ATP generation, NAD/NADH balance [44], and in the regulation of oxidative phosphorylation enzymatic chains [45]. In cardiomyocytes from healthy hearts, the close proximity of sarcoplasmic/endoplasmic reticulum to mitochondria can determine rapid changes of calcium after its release from type-2 ryanodine receptor/Ca^2+^ release channels (RyR2) and type 2 inositol 1,4,5-trisphosphate receptors (IP_3_R_2_), the most important intracellular Ca^2+^ release channels in the heart [46] (Figure 1A). Sarcoplasmic Ca^2+^ release via RyR2 and/or IP_3_R_2_ channels determines mitochondrial Ca^2+^ accumulation. Both mitochondrial Ca^2+^ excess and deficiency may result in altered mitochondrial function [47]. In particular, intracellular calcium excess can lead to mitochondrial dysfunction, with the consequence of ATP synthesis deficit and increased oxidative stress [47].

Oxidative phosphorylation in mitochondria can also be affected by altered calcium dependent protein kinases and calcineurin [48]. A physiological role of calcium in mitochondrial metabolism is evident from the effect of small levels of calcium taken up by this organelle resulting in an efficient regulation of the phosphorylation process [49]. The effects of an increased contractility with the need for highly available ATP is linked to the higher cytosolic calcium transient whose transmission to the mitochondria allows the activation of several complexes of electron transport chain [49]. The Na–Ca exchangers in the mitochondrial membrane regulate mitochondrial calcium levels and link mitochondrial calcium to intracellular sodium. In heart failure, intracellular sodium content is increased in cardiomyocytes and is closely linked to changes in mitochondrial calcium concentration that alter ROS generation, energy production, and metabolism in these organelles [50].

In the condition of heart failure where calcium cytosolic overload is observed, mitochondria are involved in opening mitochondrial permeability transition pores (mPTP) possibly to increase the mitochondria Ca^2+^ concentration. This condition conversely can trigger directly the dysfunction of those organelles resulting in cell death [51].

Another channel responsible for mitochondrial calcium handling is the calcium uniporter (mCU) [49] which allows mitochondrial calcium entrance. In adult mice, when uniporter function was blocked by the anti-estrogen drug tamoxifen, a reduction of myocardial infarction size was observed after ischemia and reperfusion [52]. A controlled calcium uptake into the mitochondria is fundamental for mitochondrial oxidative phosphorylation and ATP generation even if an excessive uptake can lead to mitochondrial dysfunction and cell death [53]. Calcium influx and efflux balance is essential for correct intracellular homeostasis and is closely regulated by influx (mCU) and efflux (Na^+^/Ca^2+^ or H^+^/Ca^2+^ exchanger or mPTP) systems [54].

## 3. Mitochondria Reactive Oxygen Species (ROS) and Reactive Nitrogen Species (RNS) Production and Calcium Handling

Cardiomyocytes can generate ROS mainly from mitochondria, where a small percentage (1–2% in physiological condition, and a higher percentage in pathological condition) of electrons do not complete the whole transfer from the series of redox reactions of the electron transport chain leading to oxygen leaks as precursors of ROS formation. Other enzymes widely present in cardiac cells—such as NAD(P)H oxidase, xanthine oxidase, and uncoupled nitric oxide synthase—participate in ROS generation [24]. In addition to ROS, there is clear evidence of an increased in RNS in heart failure experimental models and in humans [42,55,56]. Calcium regulation and ROS production are strictly interdependent [39,57]. ROS is fundamental in reshaping local and global calcium signal amplitudes and kinetics in both physiological and pathological conditions.

In physiological conditions, the activity of enzymes that organize intracellular calcium handling (SERCA) can be modified by tyrosine nitration or cysteine oxidation related to RNS generation [58,59]. Mitochondrial ROS generation, and in particular superoxide generations, are able to influence calcium microdomains at their associated dyads. An intermittent generation of superoxide by the mitochondria, named ‘superoxide flash’, in a physiological setting, clearly influences calcium sparks [60]. While superoxide flashes do not normally propagate, there is also an intermitochondrial ROS-induced ROS release occurring during pathological conditions that can burst ROS generation. Mitochondrial calcium levels (results of influx and efflux balance) regulate the aforementioned modes of mitochondrial ROS production both in constitutively or in pathophysiological settings [61]. In particular, mitochondrial ROS can affect calcium-tuning components at the levels of dyads. Under physiological conditions, spontaneous calcium spark activity is regulated by basal ROS cellular generation. The reduction of ROS generation by superoxide dismutase or reducing agents but also through the inhibition of mitochondrial complex III of electron transport chains lower the calcium spark frequency and amplitude [62].

During cardiac diseases, such as in experimental models of arterial hypertension and heart failure, as well as in heart failure patients [55,56], higher levels of myocardial and circulating ROS and RNS are reported. Such a ROS and RNS-mediated functional regulation of calcium homeostasis is bidirectional and is dependent on the quality of ROS generated, its amount, and where at the subcellular level ROS and RNS are produced.

The increased concentration of intracellular Ca^2+^ in heart failure can partially depend on the oxidation of the RyR that increases sarcoplasmic calcium leakage during diastole and reduces intrasarcoplasmic calcium content [63]. The depletion of sarcoplasmic reticulum content is also dependent on SERCA oxidation, which tends to inhibit its activity [64]. Diastolic calcium increases the susceptibility to arrhythmogenic afterdepolarizations and ventricular polymorphic arrhythmias [65].

## 4. Mitochondrial Mechanosensation and Intracellular Calcium Signaling

### 4.1. Is the Mitochondria Mechanosensitive?

**Figure 2:**
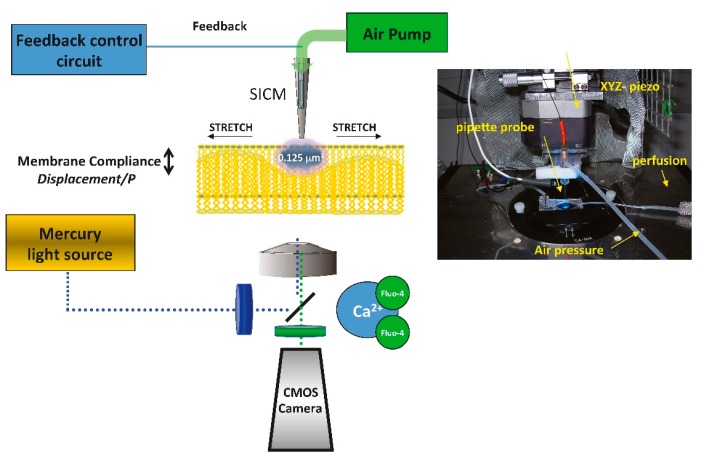
Schematic representation of nanoscopic technology for interrogates subsarcolemmal mitochondrial mechanosensitivty. Blue dotted line: excitation light. Green dotted line: emission light from the sample.

We directly interrogated subsarcolemmal mitochondria by combining scanning ion conductance microscopy (SICM) together with optical mapping of impulse propagation, with the intent to apply a hydrojet pressure via the SICM nanopipette with nanometer precision to investigate the subcellular mechanisms underlying mechanically-induced intracellular calcium release in heart failure [66] as described recently (Figure 2) [6].

**Figure 3:**
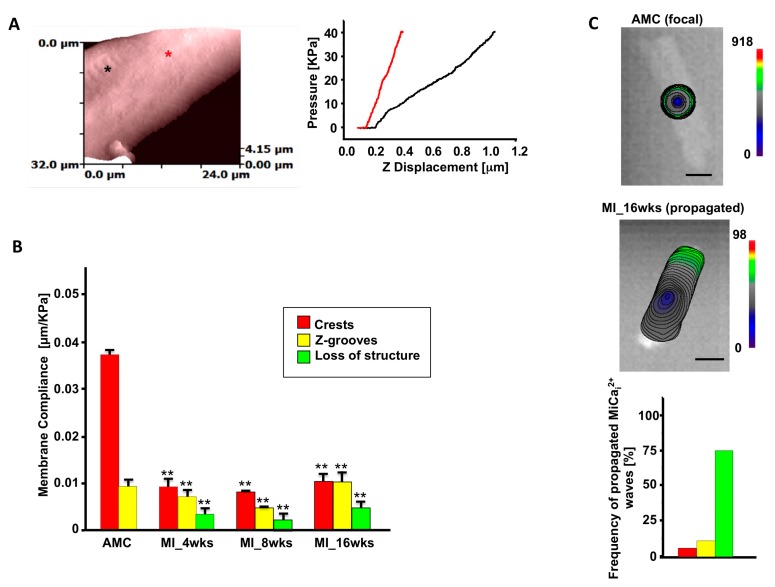
Membrane compliance related to mechanically induced Ca^2+^ release. (**A**) Left. SICM topographical image of a failing rat ventricular cardiomyocyte. Asterisks indicate the position where hydrojet pressures were applied (black = partially striated area, red = unstriated area). Right. Pressure vs. Z displacement in the two positions selected on SICM image. To note the unstriated area is stiffer; (**B**) Membrane compliance (µm/kPa) during progression toward HF (MI-16 weeks (wks)) in crest, groove, and unstriated areas; (**C**) Mechanically-induced Ca^2+^ release after hydrojet pressure delivery, in AMC cell top and in HF cell bottom. * *p* < 0.05 vs. control; ** *p* < 0.001 vs. control. Modified from [6] with permission.

Immediately, we observed that in heart failure cells—derived either from rats or human biopsies—the average Young modulus of elasticity is modulated, resulting in stiffened HF cells compared with aged match control (AMC) cardiomyocytes. Because HF cells denote unstriated areas compared to AMC cells [67] (Figure 3A) we observed that the remodeled area are stiffer in comparison with the striated ones from the same cells. We then systematically tested rat ventricular cardiomyocytes obtained at 4, 8, and 16 weeks following myocardial infarction; we found that after 4 weeks the membrane compliance (i.e., increment of stiffness) is reduced and this is a hallmark which persists for the entire progression towards HF (Figure 3B). By measuring intracellular Ca^2+^ transient during and after the release of the hydrojets, we observed that in AMC control cells the Ca^2+^ transient initiated and was confined around the mechanical stimulation site while in 16-week MI cells Ca^2+^ transient initiated and propagated in the entire cell (Figure 3C) following an abnormal cell contraction (data not shown).

### 4.2. Is the Mitochondrion a Dynamic Organelle?

Knowlton et al., demonstrate that localization of subsarcolemmal mitochondria is altered in heart failure, specifically because those organelles undergo a fission/fusion processes [68]. To monitor the position in living cells, we employed both confocal microscopy in combination with scanning ion conductance microscopy (SSCM) to investigate the sub-membrane interaction between dyads and mitochondria in failing cells compared with the gold standard technique for fixed cells, i.e., transmission electron microscopy (TEM).

**Figure 4:**
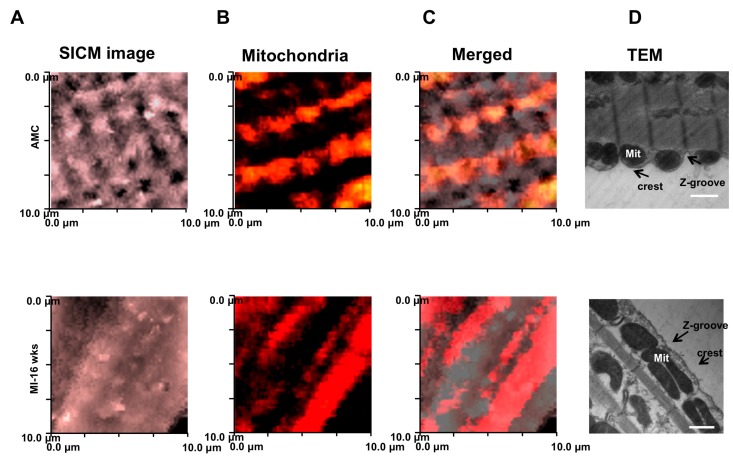
Surface scanning ion conductance microscopy analysis of mitochondrial displacement. (**A**) SICM images of 10 μm × 10 μm cardiomyocyte regions of AMC (top) and MI (bottom) cells; (**B**) Surface confocal images (obtained by SSICM) of the labelled TMRM active mitochondria in (**A**); (**C**) Merged images for SICM topography TMRM-labelled mitochondria; (**D**) Mitochondrial displacement observed by TEM. Scale bar: 500 nm Modified from [6] with permission.

In age-matched control (AMC) cardiomyocytes, TMRM-labeled mitochondria align preferentially with crests with periodic arrangements, and reflect regular arrangement of Z-grooves and T-tubule openings (Figure 4) as shown in particular on the TEM panels. We found that, in failing cardiomyocytes, mitochondria are not regularly positioned beneath the crest, but are rather fused and elongated (Figure 4B–D).

## 5. What is the Role of Mitochondrial Ca^2+^ in This Context?

**Figure 5:**
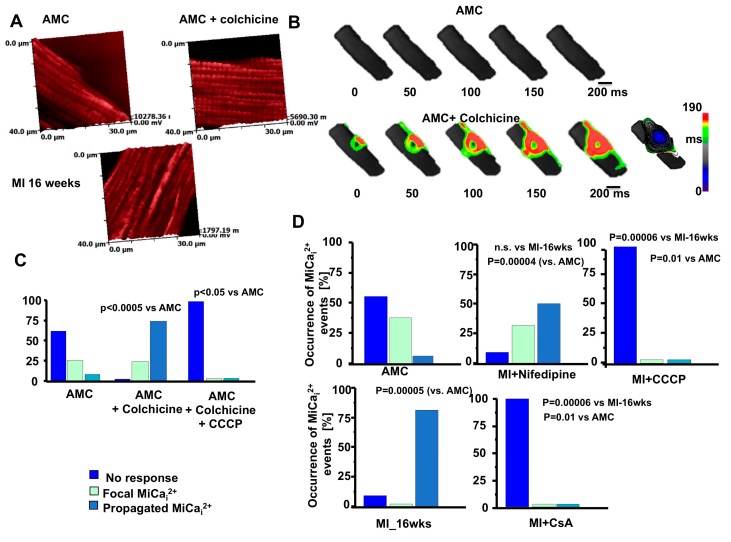
Microtubule network derangements together with mitochondrial displacement are prerequisite for mechanically induced Ca^2+^ release initiation. (**A**) TMRM-labelled mitochondria position in AMC, AMC+colchicine, and HF (MI-16 weeks); (**B**) Mechanically induced Ca^2+^ initiation in AMC cell (top) and the same cell in the presence of colchicine (bottom); (**C**) Occurrence of MiCa_i_ events in terms of no response, focal MiCa_i_ and total MiCa_i_ in AMC, AMC+colchicine, AMC+colchcine+CCCP; (**D**) Same as C for AMC, MI-16 wks, MI-16-wks + Nifedipine, MI-16-wks + CCCP, MI-16-wks + CsA. Modified from [6] with permission.

Pressure flow inducing Ca^2+^ efflux from the mitochondria has been observed by Belmonte et al. [69] independently from the calcium Ca^2+^ induced-Ca^2+^ release process. Interestingly, they observed an activation of mitochondrial Ca^2+^ induced-Ca^2+^ release both in atrial and ventricular cells. To note, this mechanical intervention does not result only in a mitochondrial Ca^2+^ efflux. Very recently, Prosser et al., were the first to demonstrate that a physiologic stretch rapidly activates reduced-form nicotinamide adenine dinucleotide phosphate (NADPH) oxidase 2 (NOX2) to produce reactive oxygen species (ROS) in a microtubules-dependent process via X-ROS signaling [65,70,71]. Based on these findings, we took in consideration not only the role of mitochondria but also their position in the cells and the microtubular organization in AMC and failing cardiomyocytes. We then started to disrupt microtubules by colchicine and visualized mitochondrial localization via SSICM. We did not observe differences between AMC cells treated with colchicine and HF cardiomyocytes, suggesting that microtubule derangement may recapitulate the HF phenotype for mechanically-induced Ca^2+^ transient (Figure 5A). Interestingly, depolymerization of the microtubules obtained by 10 µm colchicine produced a phenotype similar to the one encountered during MI (Figure 5B,C) and increased the probability of obtaining mechanically induced Ca^2+^ propagation (observed in 69% of cases). Those data are not only in agreement with those found in HF cells (mitochondrial fusion) but also with commotio cordis experiments where ventricular fibrillation was provoked with a chest impact [2].

Disruption of microtubule networks therefore enhances the likelihood of MiCa_i_ (pharmacologically by colchicine); however, during myocardial infarction, such networks are destabilized by the upregulation of the tubulin protein family (not show here) [6]. Among this, β-tubulin, which is confined to the perinuclear and interfibrillar spaces is co-localized with mitochondria [72]. The distribution [73] is limited to the external mitochondrial-containing domain, probably involving MAPs and other trans-locators. In order to identify the Ca^2+^ for MiCa_i_ we have blocked LTCC with Nifedipine (Figure 5D); however MiCa_i_ still occurred. Only by blocking mitochondrial Ca^2+^ influx and efflux, the phenomena of MiCa_i_ disappear in both AMC+colchicine and in MI-16 week cardiomyocytes.

## 6. Conclusions

In this review, we have focused on mitochondria which seem to jeopardize cardiovascular function due to their displacement/relocation and fusion in failing cardiomyocytes.

This review drew attention to the fact that mitochondria may play a more important role in a local environment. It is possible that, under certain pathological conditions, the local ROS, X-ROS, and Ca^2+^ signals could be accumulated to contribute to cardiomyocyte energy/signaling derangement.

Dissociation of excitation–metabolism coupling plus mitochondrial displacement during HF emphasizes a possible role of a ‘fine tuning’ role in cardiac physiology and pathology. Arrhythmia is a macroscale phenomenon; the mechanisms herein described (mitochondria relocation, and MiCa_i_ initiation and propagation) at single cell levels can be transposed to the mechanically induced arrhythmia encountered in vivo (triggered activity, commotio cordis, or calcium alternans) [74,75,76]. Indeed, a similar hydrojet pressure approach on in vivo beating rat hearts can induce an AV block when the jet is delivered on top of the pulmonary cone [77]. Thus, as abnormal Ca^2+^ waves underline arrhythmias, what we described here in terms of mitochondria relocation and Ca^2+^ handling may be involved as a novel arrhythmogenic substrate. Pharmacological action on cardiac mitochondria can be of upmost importance especially in the context of improving the function of a failing heart [78] when considering that HF is a bioenergetics disease. We need to take in consideration not only the organelle as such but the structural and morphological environment whereby the mitochondria is involved. Blocking the mitochondrial permeability transition pore (mPTP) was the typical example; while blocking mPTP may be beneficial during ischemic/reperfusion injury by inhibiting mitochondrial Ca^2+^ overload [79], low-dose CsA treatment (2 mg/kg/day) accelerated the development of heart failure, including dilatory LV remodeling and impaired systolic and diastolic mechanics [80]. In conclusion, we have attempted—with the aid of super-resolution based technologies—to provide novel data and open a new avenue of investigation for the cardiac microdomain involved in myocardial diseases [65,81,82]. Mechanosensing starts at the nanoscale level and SICM-based nanoscopy allows for the identification of these processes with nanometer precision.

## 7. Outlook on Mitochondria Function

Provokingly, when we talk about mitochondria—either on prokaryotic or eukaryotic cells—we always link those organelles with metabolisms. This is not the case as a recent publication in Nature demonstrates how mitochondria act as messengers between astrocytes and neurons [83]. The same heterocellular (electrical) coupling has been proposed also between myofibroblasts (i.e., the cells which populated the infarct scar) and the cardiomyocytes [84] and also between cardiomyocytes and macrophages [85]. It would be interesting to investigate if mitochondria play a role not only in the genesis but also in the perpetuation of arrhythmias.
